# Navigating Cardiovascular Risk in Type 1 Diabetes: A Comprehensive Review of Strategies for Prevention and Management

**DOI:** 10.7759/cureus.60426

**Published:** 2024-05-16

**Authors:** Shafaque Maqusood, Vivek Chakole, Sambit Dash

**Affiliations:** 1 Anaesthesiology, Jawaharlal Nehru Medical College, Datta Meghe Institute of Higher Education and Research, Wardha, IND; 2 Anesthesiology, Jawaharlal Nehru Medical College, Datta Meghe Institute of Higher Education and Research, Wardha, IND

**Keywords:** pharmacological interventions, glycemic control, management, prevention, cardiovascular risk, type 1 diabetes

## Abstract

Type 1 diabetes mellitus (T1DM) poses a significant cardiovascular risk, necessitating comprehensive prevention and management strategies. This review provides insights into the cardiovascular risk landscape in T1DM, emphasizing the importance of glycemic control, lipid management, blood pressure regulation, and lifestyle modifications. Pharmacological interventions, including insulin therapy and lipid-lowering medications, are discussed alongside lifestyle interventions such as diet, exercise, and smoking cessation. Early detection and management of cardiovascular complications are essential, highlighting the need for regular screening and multidisciplinary care. Patient-centered approaches, including shared decision-making and psychosocial support, are vital to effective care delivery. The review concludes with a call to action for healthcare providers and policymakers to prioritize cardiovascular risk management in T1DM. It explores future directions, including emerging therapies and technological innovations. By implementing evidence-based strategies and fostering collaboration across disciplines, we can mitigate cardiovascular risk and improve outcomes for individuals with T1DM.

## Introduction and background

Type 1 diabetes mellitus (T1DM) is a chronic autoimmune condition characterized by the destruction of insulin-producing pancreatic beta cells. This results in a deficiency of insulin, a hormone necessary for regulating blood sugar levels. T1DM typically develops during childhood or adolescence but can occur at any age. It requires lifelong management through insulin therapy, blood sugar monitoring, and lifestyle modifications [1]. Individuals with T1DM are at an increased risk of developing cardiovascular complications compared to the general population. These complications include coronary artery disease (CAD), stroke, peripheral artery disease, and heart failure. Despite advances in diabetes care, cardiovascular disease (CVD) remains a leading cause of morbidity and mortality in people with T1DM [2].

Given the elevated cardiovascular risk associated with T1DM, it is imperative to implement effective prevention and management strategies. These strategies aim to mitigate modifiable risk factors, optimize glycemic control, and address cardiovascular risk factors such as hypertension, dyslipidemia, and inflammation. By doing so, the incidence and severity of cardiovascular complications can be reduced, ultimately improving the overall health outcomes of individuals with T1DM [3]. The purpose of this comprehensive review is to examine the current understanding of cardiovascular risk in T1DM and to explore strategies for prevention and management. By synthesizing existing literature and highlighting recent advances in the field, this review aims to provide healthcare professionals with evidence-based recommendations for optimizing cardiovascular health in patients with T1DM.

## Review

Understanding cardiovascular risk factors in T1DM

Glycemic Control and Cardiovascular Health

Glycemic control is a pivotal factor in managing cardiovascular health among diabetes patients. Research has underscored that meticulous glycemic control can mitigate the risk of microvascular complications and yield enduring benefits concerning CVD risk, irrespective of whether patients have T1DM or type 2 diabetes mellitus (T2DM) [4]. Nonetheless, the impact of intensive glycemic control on cardiovascular outcomes remains uncertain, with specific studies suggesting a potential null or even adverse effect on these outcomes [4]. A large-scale cohort study revealed that maintaining controlled glycemia correlated with enhanced cardiovascular outcomes among diabetes patients with established CAD, particularly among those exhibiting elevated levels of the triglyceride-glucose (TyG) index, which is an indicator of insulin resistance [5]. The study noted that heightened TyG index levels associated heightened cardiovascular event risks. In contrast, controlled glycemia was linked to reduced cardiovascular event risks within the high TyG index subgroup, albeit not within the low and moderate TyG index subgroups [5]. Moreover, an additional study postulated that chronic hyperglycemia potentially contributes to coronary heart disease (CHD) in both diabetic and nondiabetic individuals, advocating for investigations into glucose-lowering strategies to mitigate heart disease risk [6]. This study unveiled a relative risk of CHD at 2.37 (95% confidence interval (CI), 1.53-3.68) among adults with diabetes, with the relative risk escalating alongside higher HbA1c levels, thus implying a positive correlation between HbA1c levels and CHD risk among adult diabetes patients [6]. Furthermore, a meta-analysis encompassing four pivotal trials illuminated that the risk reduction for combined major vascular outcomes was more pronounced among patients devoid of pre-existing macrovascular disease at baseline. This suggests that intensive glycemic control could potentially wield greater efficacy among patients in the early stages of diabetes and lacking a history of micro- or macrovascular disease [7].

Lipid Profile and Atherosclerosis

The relationship between lipid profile and atherosclerosis in individuals with T1DM is intricate and multidimensional. Studies have indicated that youth diagnosed with T1DM who maintain optimal A1C levels tend to exhibit lipid profiles that are comparable to, or even less atherogenic than, their nondiabetic counterparts. Conversely, those with suboptimal glycemic control often demonstrate elevated standard lipid levels [8]. However, it is noteworthy that youth with T1DM also display significantly heightened levels of apolipoprotein B (apoB) and a greater prevalence of small, dense LDL particles, irrespective of their glycemic control status [8]. ApoB has emerged as a superior predictor of incident CVD compared to LDL cholesterol and non-HDL cholesterol, while the presence of dense LDL particle subclasses has been linked to an elevated risk of ischemic heart disease events and arterial narrowing [8]. Therefore, the elevated levels of apoB and dense LDL particles in youth with T1DM may substantially contribute to their heightened CVD risk [8]. In patients with T1DM, advanced disturbances in lipoprotein profiles have been correlated with an increased risk of CVD [9]. Discrepancies have been observed between conventional LDL cholesterol and NMR-LDL parameters in individuals with T1DM, with a notably higher prevalence of NMR-derived LDL particle concentration (LDL-P) compared to conventional LDL cholesterol when compared to controls (38% vs. 21.1%) [9]. Certain NMR-derived LDL variables appear to provide additional insights into CVD risk assessment beyond the conventional measurements of LDL cholesterol [9]. Early detection of atherosclerosis in individuals with T1DM is paramount for preventing complications. The 2019 American Diabetes Association (ADA) guidelines advocate for intensive management of risk factors, including lipid-lowering medications (LLMs) regardless of lipid levels, blood pressure (BP) regulation, and antiplatelet agents once clinical evidence of atherosclerosis manifests [10]. However, it is concerning that the majority of patients with T1DM fail to meet all recommended targets for HbA1c, BP, lipid levels, BMI, and smoking cessation [10].

BP Management

Effectively managing BP in individuals with T1DM is paramount for mitigating the risk of CVD. These individuals' recommended target BP is below 130/80 mmHg [11,12]. Lifestyle modifications, including regular physical activity and a balanced diet, are pivotal in controlling high BP and cholesterol levels [13]. Medication may sometimes be necessary to achieve optimal BP control. Aggressive BP management in T1DM patients has been shown to reduce both micro- and macrovascular complications [14]. Angiotensin-converting enzyme inhibitors (ACEIs) are considered the first-line treatment for hypertension in individuals with T1DM, with angiotensin II receptor blockers (ARBs) being an alternative option for patients who are intolerant of ACEIs [14]. Second-line treatment options include thiazides, beta-blockers, and calcium channel blockers, and multidrug regimens are often required to attain the target BP of less than 130/80 mmHg [14]. The National Institute for Health and Care Excellence (NICE) recommends that healthcare providers emphasize the importance of maintaining BP within normal limits, offer medications when necessary, and routinely monitor BP levels [15]. Furthermore, healthcare providers should educate patients about medications' benefits and potential risks, assess treatment efficacy, and inquire about any adverse effects. Despite advancements in diabetes care, undertreatment of cardiovascular risk factors, such as dyslipidemia and hypertension, remains prevalent among individuals with T1DM, particularly among younger patients [12]. Addressing this issue necessitates harmonizing international guidelines and a deeper understanding of the barriers hindering the initiation of LLMs and antihypertensive medications (AHMs). By implementing comprehensive management strategies and addressing barriers to treatment, healthcare providers can optimize cardiovascular risk reduction in individuals with T1DM.

Inflammation and Endothelial Dysfunction

**Figure 1:**
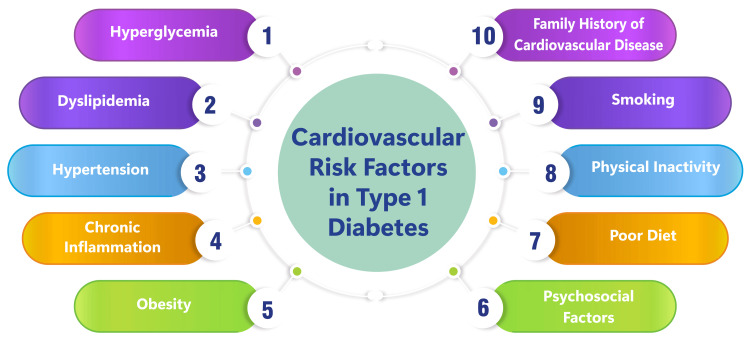
Cardiovascular risk factors in type 1 diabetes Image credit: Seema Yelne

In T1DM, inflammation and endothelial dysfunction are pivotal players in CVD genesis. Endothelial dysfunction, a hallmark of CVD, manifests as compromised endothelium function, which is a metabolically active layer lining blood vessels. This dysfunction significantly contributes to the pathophysiology of CVD in diabetes [16]. Inflammation, particularly chronic low-grade inflammation, is intricately intertwined with endothelial dysfunction and plays a central role in the genesis and progression of CVD in T1DM. Vascular dysfunction, inclusive of endothelial dysfunction, stands as a prevalent characteristic in diabetes and fosters the development of conditions such as CAD, peripheral vascular disease (PVD), and cerebrovascular disease [16]. The interplay between inflammation, endothelial dysfunction, and CVD in T1DM underscores the criticality of addressing these factors in managing and preventing cardiovascular complications in individuals with T1DM. The comprehension and targeting of inflammation and endothelial dysfunction are indispensable strategies in curtailing the excessive cardiovascular risk associated with T1DM [16]. Figure 1 shows cardiovascular risk factors in T1DM.

Prevention strategies

Lifestyle Modifications

Diet and nutrition: The dietary recommendations for individuals with T1DM revolve around maintaining optimal glycemic control, managing BP, reducing lipid levels, and fostering a healthy lifestyle through dietary choices and regular exercise. A T1DM-specific diet typically involves limiting simple carbohydrates in favor of healthier options. Whole grains such as brown rice, quinoa, and oatmeal, being rich in fiber and nutrients with a low glycemic load, are particularly recommended [17]. While fats have minimal direct impact on blood sugar, they can influence carbohydrate absorption when consumed as part of a meal. Optimal choices include plant-based fats such as olive oil, nuts, seeds, and avocado, which are associated with reduced disease risk, while animal meat fats may elevate CVD risk [17]. Protein, which provides sustained energy with minimal blood sugar impact, aids in healing and repair. Plant-based protein sources such as beans, lentils, nuts, seeds, and soy foods are preferred, along with lean animal proteins such as fish, seafood, chicken, turkey, and yogurt [17]. Incorporating protein into meals or snacks helps stabilize blood sugar levels and satiety, consequently mitigating sugar cravings [17]. Furthermore, a balanced eating plan, regular physical activity, and collaboration with healthcare professionals for insulin therapy adjustments can mitigate the risk of diabetes-related complications [18]. A T1DM diet should emphasize reasonable glycemic control, BP management, lipid level reduction, and a healthy lifestyle through diet and exercise, featuring healthful protein foods, non-starchy vegetables, nuts, seeds, beans, legumes, whole grains, healthful fats, and adequate hydration while avoiding or limiting added sugars, refined grains, processed foods, sugary beverages, fried foods, and high-fat alcoholic beverages [19].

Exercise and physical activity: Regular physical activity is paramount for overall health and well-being, irrespective of diabetes type. Exercise aids in weight management, mood enhancement, and better sleep quality [20,21]. For individuals with T1DM, balancing insulin doses with food intake and physical activity is essential. Strategic planning and understanding how blood glucose responds to exercise can prevent hypoglycemia [20,21]. Monitoring blood glucose levels before, during, and after exercise is crucial, with adjustments in insulin doses and carbohydrate intake before or during exercise to maintain safe blood sugar levels [20,21]. Various exercise modalities, including resistance training, high-intensity interval training, swimming, cycling, running, and mixed aerobic exercises, are suitable for individuals with T1DM and can mitigate CVD risk and other diabetes-related complications [22]. Barriers to physical activity, such as fear of hypoglycemia and insufficient knowledge about blood sugar management during exercise, can be overcome with proper planning, therapy adjustments, and technological aids such as continuous glucose monitoring systems and smartphone applications [23]. These advancements aim to make exercise safer and more manageable for individuals with T1DM, facilitating better glycemic control and overall health outcomes [23].

Weight management: Managing weight is a critical aspect of care for individuals with T1DM, particularly for those who are overweight or obese. Alarmingly, the prevalence of obesity among individuals with T1DM has exceeded that of the general population, with approximately 50% of patients falling into the overweight or obese category [24,25]. This trend is concerning as obesity can exacerbate insulin resistance, dyslipidemia, and cardiometabolic complications in T1DM [25]. Nutrition therapy stands as a cornerstone of weight management in T1DM. The ADA advocates for weight loss in all overweight or obese individuals with diabetes or at risk for diabetes [24]. While various nutrition-based approaches for weight loss have been explored in both diabetic and nondiabetic populations, studies specific to patients with T1DM remain limited [24]. For individuals with T2DM, specific macronutrient compositions such as low-carbohydrate or low-fat calorie-restricted diets and different eating patterns have shown efficacy in promoting weight loss [24]. However, further research is warranted to ascertain the most effective nutrition-based approach for weight loss in individuals with T1DM. Exercise also plays a pivotal role in weight management for individuals with T1DM. Increased physical activity has been linked to enhanced weight loss; however, adults with T1DM tend to engage in less physical activity than their nondiabetic counterparts due to fear of hypoglycemia [24]. To mitigate hypoglycemic risks, patients typically adjust their insulin doses before exercise, but this strategy is feasible only when exercise is planned [24]. Moreover, maintaining higher blood glucose levels before exercise to prevent hypoglycemia increases energy intake and weight gain [24]. Therefore, dynamic adjustment of insulin doses is essential during weight management in T1DM. Additionally, adjunct pharmacotherapy can be considered for weight management in T1DM. Glucagon-like peptide-1 (GLP-1) analogs, currently used to treat T2DM and obesity, have effectively reduced appetite and slowed gastric emptying, thereby facilitating weight and body fat mass reduction [24]. The use of GLP-1 analogs in T1DM is currently under investigation.

Smoking cessation: Smoking cessation holds paramount importance for individuals with diabetes, as smoking is associated with an elevated risk of diabetes-related complications, including CHD, stroke, heart failure, peripheral arterial disease, and mortality [26]. Evidence suggests that quitting smoking can lead to improvements in cardiometabolic parameters and reduced risks of cardiovascular morbidity and mortality among diabetic patients [27]. However, individuals with diabetes may encounter challenges in quitting smoking, such as concerns regarding weight gain, glycemic fluctuations, and misconceptions about pharmacotherapy for smoking cessation [28]. Hence, tailored smoking cessation support, including intensive assistance from healthcare professionals, is recommended to aid individuals with diabetes in successfully quitting smoking [28]. Research indicates that smoking cessation may initially trigger increased appetite, caloric intake, and weight gain in diabetic patients, potentially impacting glycemic control negatively [27]. Therefore, healthcare providers should prioritize preventing weight gain associated with smoking cessation in diabetic patients and offer support to address concerns and challenges related to quitting smoking [27]. Healthcare professionals must integrate smoking cessation support into diabetes management programs, provide recommendations, guidance, and treatment for nicotine dependence, and tailor drug treatment based on patient characteristics and preferences [26].

Pharmacological Interventions

Insulin therapy: Insulin therapy stands as a cornerstone in managing T1DM and has demonstrated efficacy in reducing the occurrence and progression of both micro- and macrovascular complications [29]. Intensive insulin therapy, exemplified by landmark studies such as the Diabetes Control and Complications Trial (DCCT), is pivotal for maintaining optimal blood glucose levels and mitigating complications associated with hyperglycemia, including oxidative stress, coronary calcifications, cardiac autonomic neuropathy, and impaired myocardial function [29]. Additionally, intensive insulin therapy has been associated with the concept of metabolic memory, further underscoring its role as the standard treatment for T1DM [29]. Furthermore, future therapeutic avenues for T1DM may involve GLP-based treatment strategies, with preliminary studies showing promising outcomes in reducing insulin requirements [29]. These advancements in insulin therapy and potential future treatments signify ongoing endeavors to enhance outcomes and quality of life for individuals with T1DM.

Lipid-lowering medications: LLMs constitute a crucial component of cardiovascular risk management in individuals with T1DM. Recent research indicates that despite guideline recommendations, younger individuals with T1DM receive fewer prescriptions for LLMs and AHMs compared to their older counterparts [12]. Moreover, undertreatment of cardiovascular risk factors, encompassing lipid and BP management, persists among individuals with T1DM despite advancements in diabetes care [12]. The Steno Type 1 Risk Engine, designed to predict a first cardiovascular event in T1DM, incorporates 10 risk factors, including LDL cholesterol, a well-established risk factor for CVD in the general population [30]. However, in T1DM, LDL cholesterol may not serve as an optimal marker of cardiovascular risk in primary prevention, prompting the consideration of other risk biomarkers for enhanced treatment decisions [30]. The ADA Standards of Medical Care in Diabetes recommend LDL cholesterol levels ≥ 2.6 mmol/L as indicative of increased cardiovascular risk in T1DM; however, apparently, normal serum cholesterol concentrations in T1DM often mask an atherogenic lipid profile [30]. Hence, assessing other risk factors, such as nephropathy or retinopathy, is imperative when evaluating cardiovascular risk in individuals with T1DM [30].

BP medications: Prevention strategies for CVD in individuals with T1DM) encompass pharmacological interventions, including the utilization of antiplatelets, glucose, BP, and LLMs, alongside lifestyle interventions [30]. Statin therapy is recommended for secondary prevention in T1DM patients with atherosclerotic cardiovascular disease (ASCVD), irrespective of age [30]. In cases where the risk is deemed very high, high-intensity statin therapy is advocated to reduce low-density lipoprotein cholesterol (LDL-C) levels to less than 70 mg/dL (1.8 mmol/L) using ezetimibe or a PCSK9 inhibitor if necessary [30]. The Steno Type 1 Risk Engine, designed to predict the first cardiovascular event in T1DM, integrates 10 risk factors, with age and time-weighted mean HbA1c emerging as the most significant contributors [31]. Notably, the onset of T1DM before the age of 10 is associated with a markedly elevated risk of CVD in early adulthood, particularly among women [31]. Hyperglycemia exerts a more pronounced effect on cardiovascular risk in T1DM compared to T2DM [31]. For the management of hypertension in T1DM patients, BP medications such as ACEIs and ARBs are recommended [30]. The ADA advocates for a target BP of less than 140/90 mmHg in T1DM patients with hypertension, with ACE inhibitors and ARBs demonstrating efficacy in reducing the risk of cardiovascular events and nephropathy in this population [30].

Antiplatelet agents: Antiplatelet agents represent a crucial element of cardiovascular risk management in individuals with T1DM, aimed at mitigating the risk of cardiovascular events by inhibiting platelet aggregation and blood clot formation. In T1DM patients with a known CVD, the utilization of aspirin and statin therapy is recommended to lower the risk of cardiovascular events [32]. Moreover, antiplatelet therapy is advised for individuals with a history of myocardial infarction, barring contraindications, to diminish the risk of subsequent cardiovascular events [32]. Incorporating antiplatelet agents into the comprehensive management approach for cardiovascular risk in T1DM patients, alongside interventions such as glucose control, BP management, and lifestyle modifications, underscores their significance in reducing adverse cardiovascular outcomes.

Management of cardiovascular complications

Screening and Early Detection

Screening and early detection of cardiovascular complications in individuals with T1DM are imperative due to their heightened risk of CVD. In 2016 and 2017, the prevalence of diagnosed T1DM among US adults was 0.5%, and this figure is steadily rising worldwide [33]. T1DM patients consistently face a greater total risk of CVD compared to non-diabetic individuals, with women being particularly vulnerable, as CVD becomes the leading cause of morbidity and mortality in this population [33]. A comprehensive approach beginning in childhood is essential to enhance vascular outcomes and longevity in individuals with T1DM. Investing early in the understanding and care of children with T1DM yields substantial long-term benefits in the quality of life and life expectancy [33]. The Steno Type 1 Risk Engine, designed to predict the initial cardiovascular event in T1DM, incorporates 10 risk factors, with age emerging as the most significant determinant, followed closely by time-weighted mean HbA1c [12]. The onset of T1DM before the age of 10 is associated with a 30-fold increased risk of CVD in early adulthood, with women facing a 90-fold elevated risk of acute myocardial infarction over the same period [12]. Hyperglycemia exerts a more profound effect on cardiovascular risk in T1DM compared to T2DM [12]. Recent studies suggest that up to 60% of children may exhibit cardiometabolic risk factors around puberty. By adulthood, approximately 86% of individuals may have at least one cardiovascular risk factor, with 14%-45% presenting with two or more [33]. Thus, many of these risk factors are already prevalent among youth with T1DM. Screening and early detection aim to identify high-risk individuals with T1DM and initiate appropriate interventions to mitigate CVD risk. Lifestyle interventions, such as dietary modifications and exercise, should be widely promoted and easily accessible for youth with T1DM [33].

Treatment of CAD

The treatment approach for CAD in individuals with T1DM encompasses a multifaceted strategy involving lifestyle adjustments, glucose management, BP regulation, and LLMs. The Steno Type 1 Risk Engine, designed to predict the initial cardiovascular event in T1DM, identifies 10 risk factors, with age and time-weighted mean HbA1c emerging as the most influential [12]. Notably, hyperglycemia exerts a more pronounced effect on cardiovascular risk in T1DM than in T2DM [12], thus emphasizing the pivotal role of optimal glycemic control in mitigating CVD risk. Evidence from the Diabetes Control and Complications Trial (DCCT) and its Epidemiology of Diabetes Interventions and Complications (EDIC) study underscores the efficacy of intensive insulin therapy in reducing the risk of CVD in T1DM patients [12]. Moreover, maintaining BP within the target range is crucial for minimizing CVD risk, with the ADA recommending a target BP of less than 140/90 mmHg for T1DM patients with hypertension [30]. In addition to glycemic and BP control, LLMs, notably statins, are recommended for T1DM patients with CAD, aiming to achieve a target LDL cholesterol level of less than 100 mg/dL [30]. However, it is worth noting that LDL cholesterol may not serve as an optimal marker of cardiovascular risk in primary prevention among T1DM patients [31]. As such, novel therapeutic approaches focusing on inflammation are being investigated to further reduce cardiovascular event rates in this population [31]. These advancements in treatment underscore the ongoing efforts to enhance cardiovascular outcomes and quality of life for individuals with T1DM and CAD.

Management of Hypertension

Effectively managing hypertension in individuals with T1DM is pivotal for comprehensive cardiovascular risk management. Hypertension, recognized as a significant risk factor for CVD, substantially contributes to the development and progression of atherosclerosis in T1DM patients [31]. Studies indicate that enhanced control of traditional cardiovascular risk factors, including hypertension, has resulted in remarkable advancements in survival rates among individuals with T1DM, with a notable 29% reduction in the relative risk of death observed over a decade [30]. The inclusion of systolic BP as one of the critical risk factors in the Steno Type 1 Risk Engine underscores its significance in evaluating cardiovascular risk in T1DM [33]. Hence, in the management of hypertension in individuals with T1DM, prioritizing effective BP control is paramount. This entails implementing lifestyle modifications such as a healthy diet, regular exercise, and optimal weight, complemented by pharmacological interventions when warranted. By ensuring proper hypertension management, the risk of cardiovascular complications can be mitigated, leading to improved overall health outcomes for individuals with T1DM.

Addressing Dyslipidemia

Addressing dyslipidemia in individuals with T1DM is paramount for mitigating cardiovascular risk, which stands as the primary cause of mortality among those with T1DM [12]. The Steno Type 1 Risk Engine, designed to predict the initial cardiovascular event in T1DM, encompasses 10 risk factors, with LDL-C recognized as a modifiable risk factor [34]. Current strategies for stratifying and managing cardiovascular risk in T1DM patients encompass a multifaceted approach involving glucose, BP, and LLMs alongside lifestyle interventions [34]. As recommended by the ADA, statin therapy is advocated for all patients with diabetes, including those with T1DM, aiming to reduce LDL-C levels, irrespective of baseline LDL-C levels or the presence of additional cardiovascular risk factors [12]. Alongside statin therapy, lifestyle interventions, such as dietary modifications and regular exercise, hold significant importance in diminishing cardiovascular risk among T1DM patients grappling with dyslipidemia [12]. Dietary adjustments, encompassing reductions in saturated and trans fats, coupled with increased fiber intake and the inclusion of omega-3 fatty acids, prove beneficial in lowering LDL cholesterol levels [12]. Moreover, regular exercise routines can enhance lipid profiles and reduce cardiovascular risk in individuals with T1DM [12].

Role of Anti-inflammatory Agents

The role of anti-inflammatory agents in managing cardiovascular complications in T1DM is not extensively addressed in the available search results. However, it is recognized that hyperglycemia-induced oxidative stress stands as a primary pathophysiological factor contributing to both micro and macrovascular complications in T1DM [35]. Anti-inflammatory agents hold promise in potentially mitigating inflammation and oxidative stress, thereby potentially reducing the risk of cardiovascular complications in T1DM. A study featured in Circulation highlights the pivotal role of inflammation in the onset and progression of atherosclerosis, a key contributor to CVD [12]. Elevated levels of inflammatory markers, notably C-reactive protein (CRP), were associated with heightened cardiovascular event risk in diabetic patients. Notably, the study revealed that anti-inflammatory therapy utilizing canakinumab, a monoclonal antibody targeting interleukin-1β, demonstrated efficacy in reducing the risk of recurrent cardiovascular events among individuals with atherosclerosis. Moreover, research published in Diabetes Care showcased the potential benefits of anti-inflammatory drug salsalate in alleviating inflammation and enhancing endothelial function among T1DM patients [30]. The study indicated that salsalate treatment reduced CRP levels and improved flow-mediated dilation, a marker of endothelial function, in individuals with T1DM. While these findings offer promising insights into the potential role of anti-inflammatory agents in managing cardiovascular complications in T1DM, further research is warranted to elucidate their efficacy and safety profiles comprehensively.

Patient-centered care and support

Shared Decision-Making

Shared decision-making (SDM) epitomizes a collaborative process between healthcare professionals and patients, ensuring that individuals are empowered to make decisions that resonate with their needs and preferences. In this approach, clinicians impart expertise on treatment options, evidence, risks, and benefits. At the same time, patients contribute their preferences, personal circumstances, goals, values, and beliefs, collectively deciding on their treatment [36-38]. By fostering mutual engagement, SDM enables individuals to understand the benefits, risks, and potential outcomes of various options, thereby facilitating informed decisions that align with their specific needs and preferences [37]. This patient-centered approach enhances satisfaction with care and leads to superior-quality decisions by integrating individual values, goals, and preferences with the best available evidence on treatment options [39]. As a structured framework, SDM seamlessly incorporates evidence, patient values, and preferences into medical decisions, fostering conversations that result in better-informed decisions congruent with patients' core priorities [39].

Patient Education and Empowerment

Patient education and empowerment are pivotal components of effective diabetes management, particularly for individuals navigating the challenges of T1DM, which demands a lifelong commitment to daily self-management and decision-making [40,41]. Rooted in Freire's philosophy, empowerment education underscores the holistic nature of individuals, emphasizing personal strengths and the self-generation of problems and solutions, yielding positive impacts on self-efficacy, attitudes, and metabolic control among patients with T1DM [40]. Central to this approach is establishing a collaborative relationship between patients and healthcare providers, wherein patients assume an active role in their care, leveraging their knowledge, resources, and experiences to make informed decisions [40]. This empowerment-driven approach has significantly enhanced health outcomes and quality of life, particularly in young individuals grappling with chronic illnesses such as T1DM [40].

Diabetes self-management education (DSME) and support (DSMS) emerge as instrumental strategies in fostering patient engagement and active participation in care, serving to avert acute complications and ultimately enhance long-term outcomes and quality of life for individuals with diabetes [41]. By furnishing patients with essential information, skills, and self-efficacy, DSME and DSMS empower individuals to seamlessly integrate self-management into their daily lives and shoulder responsibility for their care [41]. Notably, empowerment education tailored for teenagers with T1DM yields positive outcomes in disease management, healthcare utilization, mental health, and overall quality of life [40]. Critical elements of effective empowerment education for young individuals encompass recognizing youth as valuable resources, fostering group cohesion through dialogue, and engaging them as decision-makers in social endeavors [40]. Additionally, tools such as the "Check your health" instrument play a crucial role in evaluating the effectiveness of empowerment education interventions among young individuals with T1DM, offering insights into perceived physical and mental health, social relations, overall quality of life, and the perceived burden of diabetes [40].

Psychosocial Support

Psychosocial support emerges as a critical aspect of managing T1DM and its associated cardiovascular risks. Diagnosing T1DM can instigate significant stress for patients and their families, necessitating a profound adjustment to the myriad of daily tasks such as blood glucose monitoring, injections, and carbohydrate tracking [42]. Adolescence presents a unique challenge, with biological and socioeconomic changes often leading to emotional turmoil, depression, or anxiety [42]. Psychosocial factors wield considerable influence over the etiology, pathogenesis, and treatment of T1DM, with psychiatric disorders, particularly internalizing behavioral issues such as depression, being identified as risk factors [42]. A study exploring family support, anxiety, depressive symptoms, and their impact on glycemic control in children with T1DM revealed a dearth of awareness regarding the psychological toll of chronic illnesses among healthcare professionals, patients, and caregivers alike [42]. Hence, providing psychosocial support to patients and their families is paramount to help them navigate the stress and emotional upheaval linked with T1DM. The ADA advocates for routine screening of depressive symptoms in diabetic patients, given that elevated depressive symptoms and depressive disorders afflict approximately one in four patients with T1DM or T2DM [43]. Providers are urged to consider annual depression screenings for all diabetic patients and those with a self-reported history of depression.

Furthermore, screenings should commence at the diagnosis of complications or when significant changes in medical status occur [43]. Referrals for depression treatment should be directed to mental health professionals proficient in cognitive behavioral therapy, interpersonal therapy, or other evidence-based approaches, with collaborative care with the patient's diabetes treatment team being essential [43]. Beyond depression, other psychological comorbidities, such as anxiety disorders, eating disorders, and insufficient social and familial support, can impede diabetes self-management and warrant attention [43]. Hence, routine monitoring and screening for diabetes distress, depression, anxiety, eating disorders, and adequate social and familial support are indispensable components of diabetes management [43]. Additionally, addressing contextual factors that hinder care implementation is imperative for optimizing patient outcomes and well-being.

Emerging research and future directions

Novel Therapeutic Approaches

Novel therapeutic approaches for managing cardiovascular risk in T1DM encompass a spectrum of innovative strategies, including nanotechnology-based insulin delivery systems, microRNA, and stem cell therapy. Additionally, there is a growing recognition of the underutilization of statins in patients with T2DM, highlighting a potential avenue for optimizing cardiovascular risk management [44]. Elderly patients with diabetes mellitus are receiving increased attention, with a particular focus on frailty, aiming to tailor care to their unique needs. Furthermore, investigations into the interplay between diabetes, smoking, periodontal parameters, and salivary matrix metalloproteinase-8 levels shed light on novel avenues for comprehensive diabetes management [44]. The armamentarium of novel therapeutic agents for diabetes mellitus continues to expand, with promising candidates such as pramlintide, incretin-based therapies, and SGLT-2 inhibitors demonstrating potential benefits in glycemic control and cardiovascular risk reduction [44,45]. A recent study delved into time trends of cardiovascular risk management in T1DM using real-life data from a nationwide diabetes register. Encouragingly, the study revealed decreasing HbA1c, LDL-C, and BP levels alongside a declining proportion of smokers and individuals with glycemic dysregulation, dyslipidemia, and hypertension from 2010 to 2017 [46]. However, the study also underscored lingering challenges, with one-fifth of the T1DM population exhibiting severe dysregulation (HbA1c > 75 mmol/mol) and one-fourth demonstrating an eGFR < 60 mL/min/1.73 m^2^ by January 1, 2017 [46]. These findings emphasize the imperative to innovate and optimize care strategies for individuals with T1DM to effectively mitigate cardiovascular risk.

Precision Medicine in T1DM and Cardiovascular Risk

The concept of precision medicine in T1DM represents a paradigm shift towards personalized treatment strategies tailored to individual characteristics, with the overarching goal of achieving curative outcomes while minimizing adverse effects [47]. Precision medicine encompasses a multifaceted approach that involves early identification of at-risk individuals, classification into subgroups based on unique characteristics, and optimization of treatment regimens to mitigate the risk of complications in those with T1DM [47]. Central to this approach is the recognition of individual variability in disease progression and treatment response, highlighting the need for tailored interventions that address each patient's specific needs and risk factors [47]. In the context of cardiovascular risk management in T1DM, precision medicine holds significant promise for enhancing risk prediction and optimizing therapeutic interventions [48]. Despite the absence of traditional cardiovascular risk factors such as dyslipidemia or hypertension, individuals with T1DM remain predisposed to cardiovascular complications, including heart failure [48]. Precision medicine offers a means to identify those at heightened risk of developing such complications and tailor personalized interventions to effectively mitigate these risks [48]. By leveraging a comprehensive array of biological, behavioral, and environmental data for precision monitoring, healthcare providers can gain deeper insights into an individual's disease trajectory, treatment response, and overall health status [49]. This holistic approach enables clinicians to optimize care delivery, anticipate potential complications, and implement targeted interventions to prevent adverse outcomes [49]. Ultimately, precision medicine in T1DM represents a transformative approach that empowers healthcare providers to deliver individualized care, improving patient outcomes and quality of life.

Technological Innovations for Monitoring and Management

Technological advancements have revolutionized the management of T1DM in recent years, offering patients new tools for improved glycemic control and quality of life [50,51]. Insulin pumps, a cornerstone of modern diabetes management, provide greater flexibility in insulin delivery compared to traditional injection methods [50]. Concurrently, continuous glucose monitoring (CGM) systems have emerged as invaluable tools for maintaining stable glycemic levels, reducing the incidence of hypoglycemia and severe hyperglycemia, and improving overall glycemic control and HbA1c levels [50,52]. Despite their benefits, CGM systems may present challenges such as higher costs, the need for periodic finger prick glucose tests for calibration, and the potential for invasiveness or limited device lifespan [50,52]. However, the integration of CGM with insulin pump technology has paved the way for automated insulin delivery (AID) systems, also known as artificial or bionic pancreas systems [53]. These innovative systems automatically utilize mathematical algorithms to adjust insulin delivery based on real-time glucose monitoring data, offering features like threshold suspend and predictive low glucose suspend to prevent hypoglycemia [53]. Hybrid-closed-loop systems, which modulate insulin delivery with user input, and fully closed-loop systems, which operate independently of user input, represent significant advancements in AID technology [53]. Additionally, bihormonal systems, incorporating insulin and an additional hormone-like glucagon, show promise for achieving superior glycemic control compared to insulin-only systems [53]. Studies have demonstrated that these technologies can improve glycemic outcomes and reduce disease burden for patients and their families [53]. However, further research is needed to elucidate the long-term benefits of these devices on glycemic control, quality of life, and overall health outcomes in individuals with T1DM [53]. Despite the ongoing challenges and uncertainties, technological innovations play a pivotal role in enhancing the management and care of T1DM patients, offering hope for a brighter future in diabetes management.

## Conclusions

In conclusion, individuals with T1DM are confronted with an elevated risk of cardiovascular complications, necessitating proactive prevention and management strategies. This review has underscored the significance of optimizing glycemic control, addressing modifiable risk factors such as hypertension and dyslipidemia, promoting lifestyle modifications, and employing pharmacological interventions where appropriate. Healthcare providers must remain vigilant in assessing cardiovascular risk factors in patients with T1DM and providing comprehensive care to mitigate these risks. Furthermore, policymakers must prioritize initiatives aimed at enhancing access to diabetes care, fostering healthy lifestyles, and supporting research endeavors focused on cardiovascular risk management in this population. The future of cardiovascular risk management in T1DM holds promise with ongoing research and technological advancements. Novel therapies targeting underlying disease mechanisms, precision medicine approaches tailored to individual patient profiles, and integrating digital health technologies for remote monitoring and personalized care delivery offer potential avenues for improving outcomes. By leveraging these advancements and fostering collaboration across disciplines, we can strive towards a future where individuals with T1DM can lead longer, healthier lives free from the burden of cardiovascular complications.
